# O4-7 An alternative framework for HEPA projects: Developing recommendations for the use of the Capability Approach

**DOI:** 10.1093/eurpub/ckac094.031

**Published:** 2022-08-29

**Authors:** Maike Till, Peter Gelius, Thomas Abel

**Affiliations:** 1 Department of Sportscience and Sport, Friedrich-Alexander-Universität Erlangen-Nürnberg, Erlangen, Germany; 2 Institute of Social and Preventive Medicine, University of Bern, Bern, Switzerland

**Keywords:** Framework Development, Physical Activity Promotion, Participatory Research

## Abstract

**Background:**

Using Amartya Sen's capability approach (CA) to conceptualize physical activity (PA) promotion projects has been suggested as a promising alternative to conventional theories, as it focuses on the real opportunities people have to engage in PA rather than on PA behavior alone. The Capital4Health research consortium used the CA to conceptualize and implement PA projects in four different settings across the life-course. The aim of the study was to evaluate the implementation of the CA in these projects and to develop recommendations for the use of the concept in future PA promotion projects.

**Methods:**

Based on an overarching analytical framework, we investigated the utilization of the CA in the individual projects using document analysis, workshops, and group interviews with project teams. Results were used to develop a set of draft principles and recommendations for effectively employing the CA in future projects and as a bridging framework for larger consortia. A participatory process combining elements of action research and Delphi surveys is currently conducted with all members of the Capital4Health consortium to arrive at final set of agreed-upon principles.

**Results:**

Preliminary results show that the use of the CA varied substantially between projects and settings, but that a number of common conclusions can be drawn. A future framework for using capabilities for PA promotion may focus on three areas: (a) Project conceptualization should address both the target group and relevant multipliers. It should also consider both individual PA competences and structural factors, (b) Evaluation should cover both capabilities for PA as well as actual changes in PA levels to assess health impact on multiple levels, (c) The CA may be very useful to research consortia for developing shared goals and evaluation frameworks. This, however, requires a shared knowledge base and agreement about central theoretical concepts.

**Conclusions:**

The CA constitutes a potentially useful theoretical basis for both individual PA promotion projects and multi-setting research consortia. However, the application of the concept is complex and may vary significantly between settings. The proposed guiding principles may therefore provide a useful aid to future projects wishing to apply this innovative approach.

